# Interactions among tumor subtype, PPARγ expression, and adipose proliferation shape outcomes in breast cancer

**DOI:** 10.14814/phy2.70649

**Published:** 2025-11-14

**Authors:** Aditya Shah, Katie Liu, Ryan Liu, Gautham Ramshankar, Curtis J. Perry, Rachel J. Perry

**Affiliations:** ^1^ Departments of Cellular & Molecular Physiology, Internal Medicine (Endocrinology), and Comparative Medicine Yale University New Haven Connecticut USA; ^2^ Woodbridge Academy Magnet School Woodbridge New Jersey USA; ^3^ Cedar Park High School Cedar Park Texas USA; ^4^ Brown University Providence Rhode Island USA; ^5^ Yale Cancer Center New Haven Connecticut USA; ^6^ Department of Internal Medicine (Hematology/Oncology) Yale University New Haven Connecticut USA

**Keywords:** adipose tissue, breast neoplasms, fluorothymidine F18, lipids, peroxisome proliferator‐activated receptor gamma

## Abstract

Breast cancer progression is influenced by tumor subtype, metabolic environment, and patient factors, including menopausal status and BMI. In this study, we utilized publicly available data to investigate the prognostic relevance of *PPARγ* gene's expression across different subgroups. We also examined adipose tissue proliferation in patients with various tumor subtypes and phenotypic cohorts. We analyzed RNA‐seq data from primary breast cancer patients in the TCGA‐BRCA cohort, stratifying patients by *PPARγ* expression, menopausal status, and tumor receptor subtype. Kaplan–Meier analyses revealed that high *PPARγ* expression was associated with improved overall survival, particularly in premenopausal patients. Complementing this, we analyzed PET‐CT scans from breast cancer patients in the ACRIN‐6888 trial, focusing on standardized uptake value (SUV) metrics of a cell cycle tracer, 3′‐deoxy‐3′‐[18F]‐fluorothymidine (^18^F‐FLT) in visceral and subcutaneous adipose tissue. Postmenopausal patients had lower visceral adipose tissue SUV_mean_, and patients with ER+ or non‐TNBC tumors showed lower SUV_peak_ and SUV_max_ of both adipose tissue types, indicating metabolic/proliferative reprogramming of adipose tissue based on tumor subtype. We hypothesize that PPARγ expression and adipose proliferation differentially affect survival across subtypes and menopausal status, providing deeper insight into PPARγ as a therapeutic target in breast cancer and the potential implications for precision medicine treatments.

## 1 INTRODUCTION

Breast cancer is the most prevalent form of cancer affecting women–it accounts for one‐eighth of all cancers diagnosed, with the number of breast cancer diagnoses consistently increasing in recent years (Arnold et al., 2022). Obesity–which is accompanied by alterations in metabolism–is a key risk factor for breast cancer in a subtype‐dependent manner: obesity increases the risk of triple‐negative breast cancer by 36%, but luminal subtype A by a non‐significant 17% and does not increase the risk of other subtypes (Sudan et al., 2024). Clearly, breast cancer cases are heterogeneous: understanding more about different mechanisms of breast cancer by patient subpopulations, and its corresponding metabolism, will help pave the way for personalized medicine and more effective therapies (Cho et al., 2012; Guo et al., 2023). Due to this, it is becoming increasingly important to understand the specific metabolic mechanisms that exacerbate the disease. Otto Warburg pioneered the idea that, due to their need for increased energetic input to continue to proliferate, rapidly dividing cells exhibit a marked increase in glucose uptake (Warburg, 1925). Researchers have continued exploring the central role of glucose metabolism in cancer. However, other metabolic pathways essential for tumor growth have often been overlooked, including lipid metabolism, the focus of this study. In addition to the classic Warburg effect, where tumor cells themselves rely on aerobic glycolysis, more recent work has described a so‐called Reverse Warburg Effect. In this model, adipocytes and stromal fibroblasts within the adipose‐rich tumor niche can provide metabolic substrates that synergize with lipid pathways, further linking systemic adipose metabolism to tumor progression.

Besides the genetic predispositions that contribute to the risk of cancer onset, environmental factors and behaviors can strongly shape and reduce risk. For example, physical exercise reduces fat volumes throughout the body and around tumors, with a specific impact on visceral and subcutaneous fat during cancer development (Spanoudaki et al., 2023). The typical milieu in which tumors arise is likely also important, as breast cancers develop in lipid‐rich microenvironments. This unique environmental association between lipid metabolism and breast cancer makes it particularly interesting to study Peroxisome proliferator‐activated receptor gamma (*PPARγ*) gene's prominence and potential promise as a biomarker in breast cancer (Li et al., 2024). Playing a role in adipocyte differentiation, *PPARγ* is highly expressed in adipose tissue and widely regarded as a master regulator of lipogenesis. Furthermore, reductions in mitochondrial function in cancer have been strongly associated with tumor proliferation–*PPARγ* regulates mitochondrial biogenesis and oxidation by promoting *PCG‐1α* (*PPARGC1A*) transcription, thereby enhancing ATP production from fatty acids. The key metabolic hallmark of cancer is a transition from oxidative to glycolytic metabolism (Li et al., 2024; Zheng, 2012). Therefore, it is likely that higher expression of genes that promote oxidative metabolism, such as *PPARγ*, may be protective against tumor progression. The impact of lipid metabolism‐related genes, and their interaction with other clinical characteristics, is of particular interest when studying breast cancer, because it is a tumor type that arises in an adipose microenvironment.

Menopausal status is a strong modulator of fat distribution and metabolism, with significance for both breast cancer risk and progression (Surakasula et al., 2014). During menopause, declining hormonal levels (primarily estrogen) shift fat from a predominantly subcutaneous fat distribution to a more visceral fat distribution, increasing central adiposity, but also altering the metabolic environment. It is particularly interesting to study how patients with different tumor types can be affected during menopause: hormone receptor‐positive (HR+) tumors are driven by adipose‐derived molecules, making them more common in women with higher adiposity, and perhaps for this reason also in postmenopausal women (Bhardwaj & Brown, 2021; Dimauro et al., 2021). On the other hand, Human Epidermal Growth Factor Receptor 2 negative (HER2‐negative) tumors are driven more commonly by inflammatory cytokines and metabolic pathways, but can be affected by obesity status and lipid metabolism, making it relevant to study how prognosis can differs among patients with different tumor types (Sparano et al., 2022). Triple‐negative breast cancer (TNBC) tumors are also fueled by immune dysregulation and heightened glycolytic metabolism, and are even more often found in patients with metabolic dysfunction as well (Naik & Decock, 2020; Sun et al., 2020). It is important to note that visceral fat is particularly active in releasing inflammatory cytokines which can contribute to tumor progression as well as insulin resistance, among other problems (Zatterale et al., 2019).

PPARγ is further interesting to study in relation to menopausal status, as the reduction in estrogen that occurs with menopause removes a regulatory influence on adipocyte function and lipid metabolism, allowing lipogenesis and fatty acid oxidation pathways, such as those regulated by PPARγ to become more prominent (Kim & Ko, 2023; Marsh et al., 2023). This makes postmenopausal women particularly susceptible to cancer thriving on lipid metabolism, and developing a further understanding of how PPARγ's activity is influenced by menopausal status can provide insight into developing targeted therapies for breast cancer prognosis across tumor subtypes. To study these relationships, we utilized a multi‐modal approach, performing analysis on Positron Emission Tomography–Computed Tomography (PET‐CT) scans from The Cancer Imaging Archive (TCIA), and analyzing the prognostic impact of *PPARG* gene expression in the TCGA breast cancer cohort (ACRIN‐FLT‐BREAST, 2015; Clark et al., 2013; Goldman et al., 2020; Peck et al., 2015; Sanghera et al., 2014). PET‐CT imaging is widely used in clinical practice to assess metabolic and proliferative activity in tumors and by using radiotracers, such as ^18^F‐FLT; using these scans, we are able to quantify proliferation rate in tissue, which is particularly relevant in oncology due to metabolic reprogramming (Chi et al., 2021; Hernandez‐Quiles et al., 2021). In this work, we measure the standard uptake values (SUV) of ^18^F‐FLT in both visceral and combined (subcutaneous and visceral) adipose tissue, to understand adipose tissue proliferation in breast cancer patients, and in turn to further contextualize findings from our genomic analyses. After analyzing patient scans and transcriptomics data, we further segmented the data by both menopausal status and BMI for the PET‐CT image analyses and Kaplan–Meier survival analyses. While PPARγ has been implicated in lipid metabolism and adipocyte regulation, its role as a prognostic biomarker across breast cancer subtypes—particularly in relation to menopausal status and adipose metabolic activity—remains insufficiently understood. Furthermore, the interplay between systemic adipose metabolism (measured via PET‐FLT) and transcriptomic regulation has not been fully addressed in prior studies. These results add to our understanding of breast cancer, paving the way for precision medicine therapies targeting these adipocyte‐based pathways.

## 2 RESULTS

### 2.1 Low *PPARγ* expression typically reduces survival across cohorts

**FIGURE 1:**
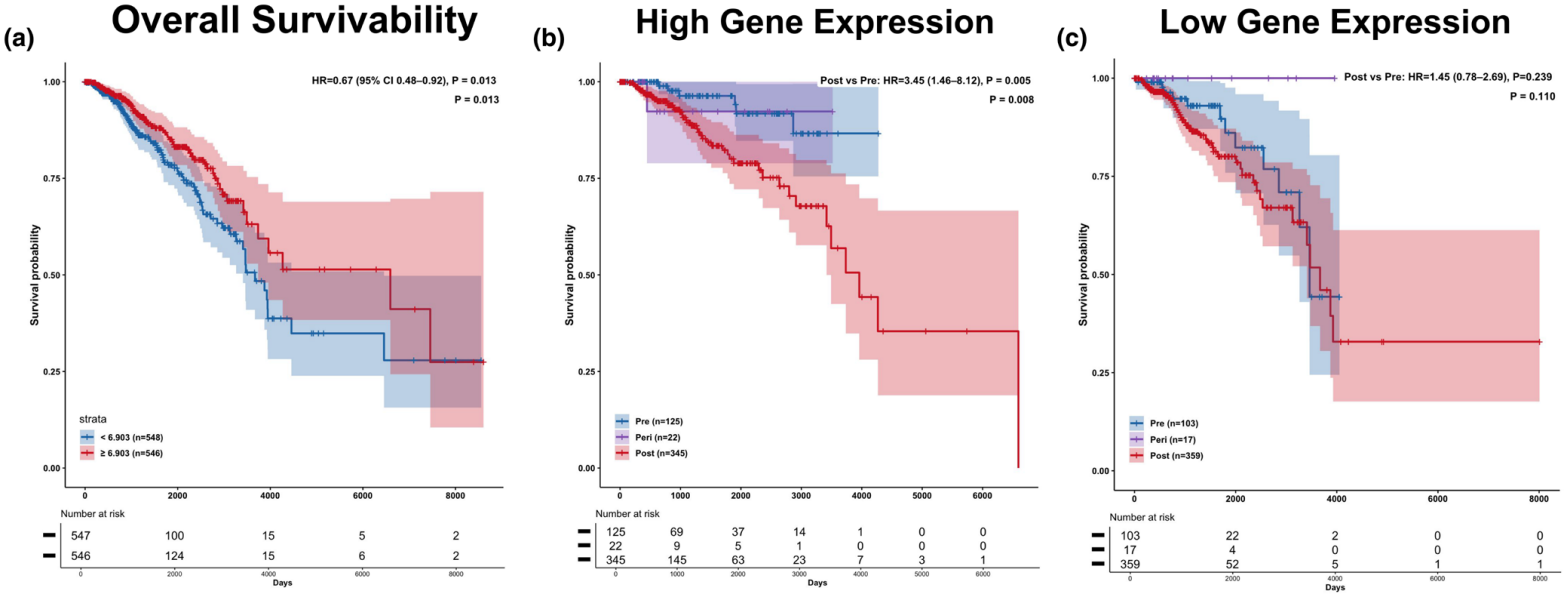
Low *PPARγ* expression significantly reduces survivability across TCGA BRCA patients & expression significantly changes survival rate in premenopausal patients. (a) Overall survivability of patients with low *PPARγ* expression (<6.903) in TCGA cohort is consistently lower than those with high expression for 8605 days after initial diagnosis (*p* < 0.05). (b) In patients with high *PPARγ* expression (≥6.903), postmenopausal patients had significantly lower survival, with all postmenopausal patients in the cohort passing away after ~6500 days. Those with premenopausal or in peri‐menopause had significantly better survivability outcomes as shown in the data even ~4000 days after initial diagnosis, hinting at *PPARγ*'s high expression playing a role in patient survivability during perimenopausal and premenopausal periods. (c) In patients with low *PPARγ* expression (<6.903), there is not as clear of a relationship between survivability and menopausal status, suggesting low *PPARγ* expression hinders some survivability function that is not specific to menopause, or possibly age.

**FIGURE 2:**
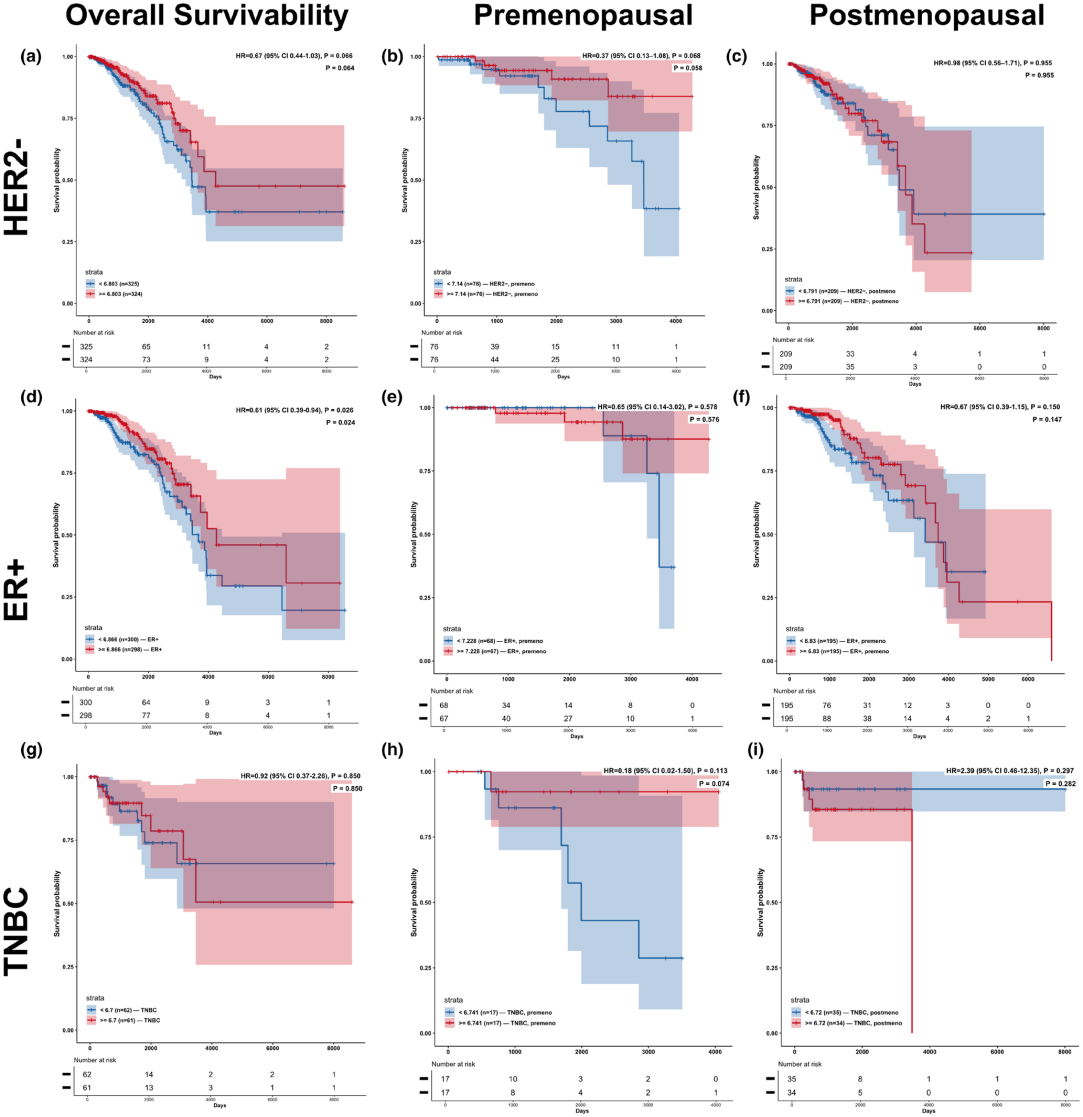
*PPARγ* expression significantly impacts survival in ER+ patients, and the postmenopausalTNBC cohort exhibits an opposing pattern. (a) Overall survival in patients with HER2‐negative tumors shows the same trend generalized to all primary tumors–those with higher *PPARγ* expression have higher survival rates  (b) Overall survivability in premenopausal patients with HER2‐negative tumors show the same trend as those with primary tumors; while there seems to be no notable difference in the first ~2000 days, there is a steep difference thereafter. (c) Overall survivability in postmenopausal patients with HER2‐negative tumors shows the same relationship through ~4000 days, and a weak difference thereafter. (d) Overall survival in patients with ER+ tumors also show the same trend as the overall cohort, those with higher expression consistently show better survival. (e) Overall survivability in premenopausal patients with ER+ tumors show the same trend and a steeper decrease in survivability for low‐expression patients after ~3000 days. (f) Overall survivability in postmenopausal patients with ER+ tumors shows minimal differences until ~4000 days, when patients with high‐expression tend to lower survival (g) Overall survival in the TNBC cohort shows a different trend as patients with higher prognosis have a tendency for a lower survival rate after ~3000 days; however, results are not statistically significant. (h) The premenopausal TNBC cohort shows the trend consistent with the full cohort, and different from the TNBC overall data: patients with higher *PPARγ* expression show a marginally significant increase in survival. (i) Postmenopausal patients with TNBC tumors show the trend characteristic to TNBC tumors, as patients with high *PPARγ* expression have lower survival across the entire timeline, although these results are underpowered due to low sample size.

Across our data, by comparing patients with low tumor *PPARγ* expression versus high *PPARγ* expression, we find that typically patients with low expression have worse survival. Across the entire TCGA BRCA cohort, from 1094 patients, we find that patients with high *PPARγ* expression (≥6.903) have higher overall survival throughout the entire duration of study (8000+ days), with significance (Figure 1a). In the HER2‐negative patient cohort, we again find the same trend to be true with significance, as throughout the entire timeline, patients with high *PPARγ* expression (≥6.866), have higher overall survival, indicating that the trend remains similar in patients without HER2 protein in their tumors (Figure 2a). In a contrasting cohort where patients are positive for a receptor type, the ER+ cohort, the same trend holds as patients with high *PPARγ* expression (≥6.803), again have higher survival in the entire cohort (Figure 2d). Alternatively, in the triple‐negative breast cancer cohort, we find an opposing trend in the long term–initially up to ~3500 days, the typical trend is seen, where patients with high *PPARγ* expression (≥6.700) tend to exhibit higher survivability; however, after 3500 days, patients with high *PPARγ* expression have a trend toward lower survival (Figure 2g), suggesting that high *PPARγ* levels may contribute to long‐term outcomes in patients with TNBC. However, these results were not statistically significant and should be considered more suggestive than conclusive.

### 2.2 *PPARγ* expression correlates with menopausal status and breast cancer survival outcomes

When analyzing data from the entire cohort, and segmenting groups based on menopausal status, we find that premenopausal patients have higher survival when compared to postmenopausal patients. This distinction is more clear in high *PPARγ* expression cohorts, where premenopausal patients continuously had higher survival rates, with statistical significance (Figure 1b). While the same trend is true in the low *PPARγ* expression cohort, it is not significant, and even in the long term nearing 3500 days, postmenopausal patients have a higher survivability rate for ~500 days (Figure 1c). In the premenopausal cohort, across tumor receptor types (HER2‐negative, ER+, TNBC), patients with low *PPARγ* expression have lower survival, especially in the ER+ and TNBC cohorts where there is marginal significance; in the HER2‐negative cohort, there are minimal differences across the two groups (Figure 2b,e,h). In the postmenopausal group the differences are not as clear, especially in the ER+ group where no differences can be seen until ~4000 days, where patients with high *PPARγ* expression have lower survival (Figure 2f). The same trend is consistent in the postmenopausal cohorts of patients with HER2‐negative and TNBC tumors, as patients with high *PPARγ* expression have lower survival (Figure 2c,i).

### 2.3 Adipose tissue SUV metrics vary by menopausal status and tumor receptor status

**FIGURE 3:**
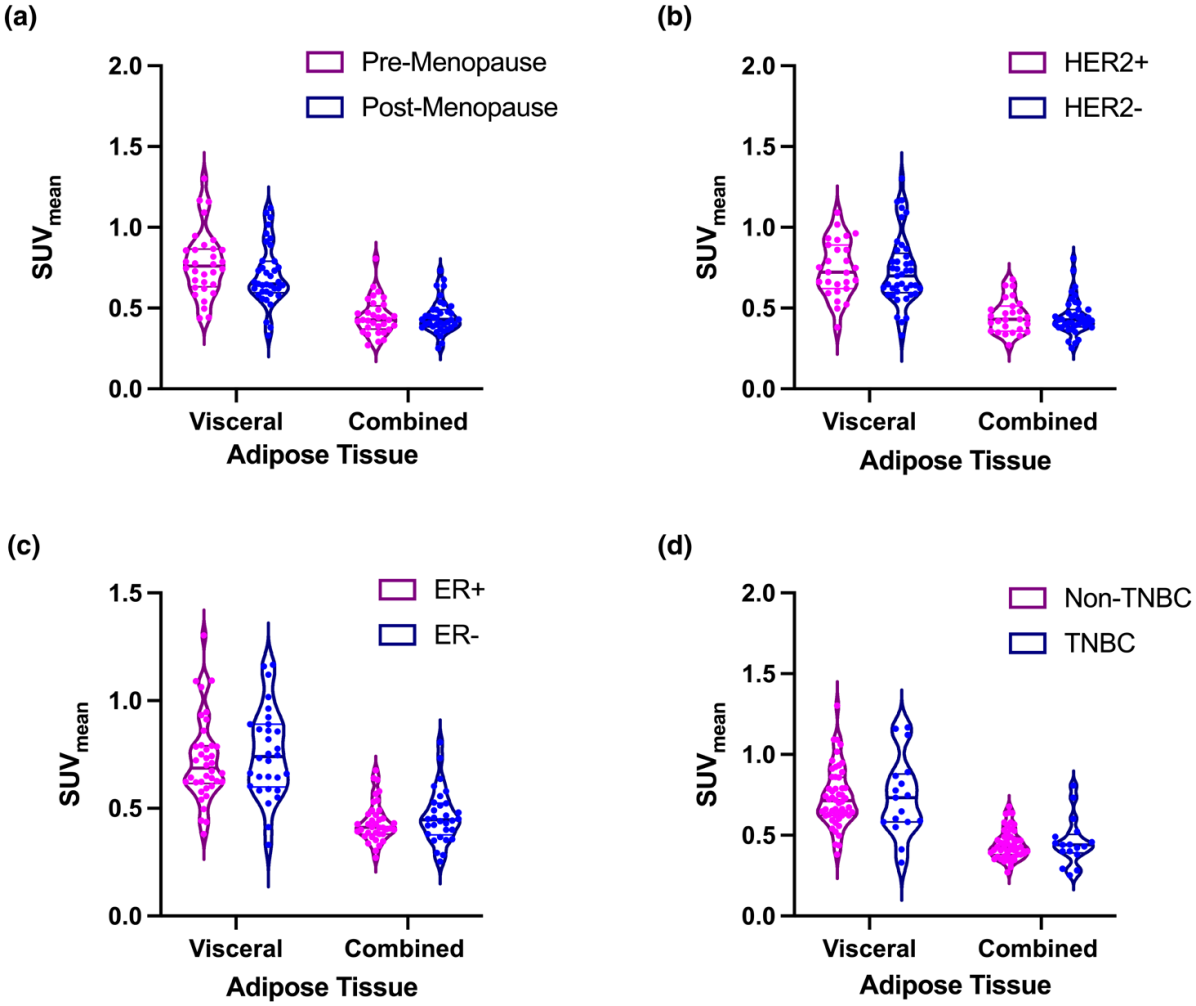
Adipose tissue SUV_mean_ remains consistent across tumor types and Menopausal States in breast cancer. (a) Visceral adipose tissue SUV_mean_ shows a decrease in postmenopausal (*N* = 37) patients compared to premenopausal patients (*N* = 32), while combined adipose tissue SUV does not show any changes, indicating differential metabolic uptake mechanisms in visceral tissue. (b) Visceral and combined adipose tissue SUV_mean_ show very slight decreases in SUV_mean_ in patients with HER2‐negative tumor types (*N* = 41) vs. patients with HER2+ tumors (*N* = 27); (c) Patients with ER+ tumors (*N* = 38) have decreased SUV_mean_ for both visceral and combined adipose tissue compared to patients with ER‐ tumors (*N* = 30), indicating decreased metabolic activity in fat tissue. (d) Patients with TNBC tumors (*N* = 17) show a slight increase in visceral adipose tissue SUV_mean_ and a combined adipose tissue SUV_mean_ compared to patients without TNBC tumors, however differences are not statistically different.

**FIGURE 4:**
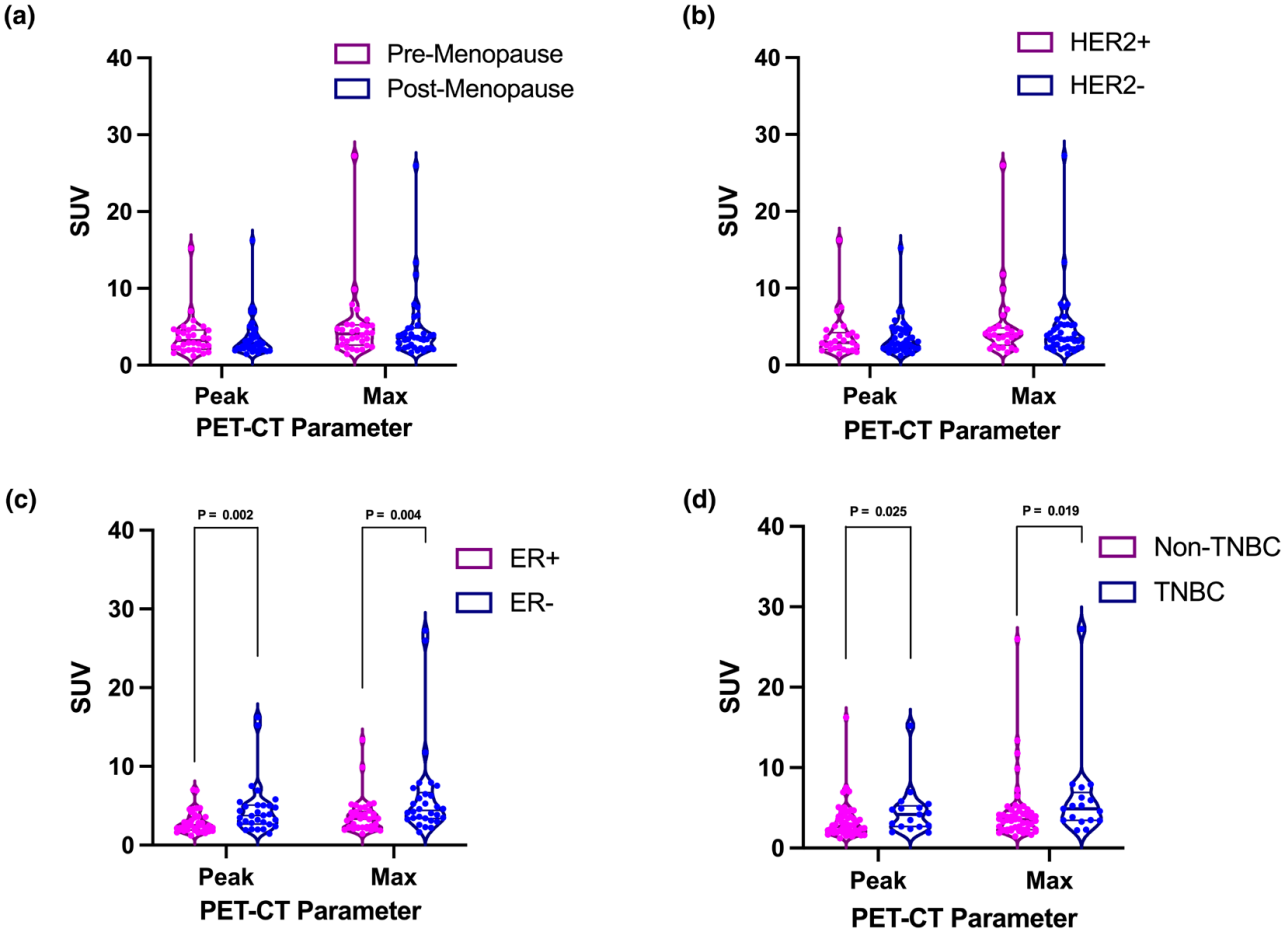
Standard uptake value metrics (g/mL) of adipose tissue differ significantly for ER+ and TNBC tumor types. (a) SUV_peak_ and SUV_max_ show no difference based on menopausal status, with a negligible decrease in the postmenopausal cohort for both tissue types. (b) HER2‐negative tumors also show a slight decrease in SUV metrics compared to HER2+ tumors, but the differences are not significant. (c) ER+ tumors show significantly lower SUV_peak_ and SUV_max_ in adipose tissue when compared to ER‐ tumors. (d) Patients with TNBC tumors show significantly higher SUV_peak_ and SUV_max_ in adipose tissue versus patients with any positive receptor tumor types.

Across cohorts, both visceral and combined adipose tissue SUV_mean_ do not significantly differ between any cohort versus its given alternative (i.e., premenopausal vs. postmenopausal) (Figure 3a). Similarly, patients who were hormone receptor‐positive for a given receptor did not exhibit differences in SUV_mean_ for either VAT or SAT as compared to patients with hormone receptor‐negative tumors (Figure 3b–d). On the other hand, SUV_peak_ and SUV_max_ showed significant differences in the TNBC and ER tumor comparisons, and the trend was similar to that found with the SUV_mean_ analyses–patients whose tumors were HR+ had lower SUV values (Figure 4c,d); comparisons of SUV_peak_ and SUV_max_ between menopausal cohorts and HER2‐negative cohorts showed no significant differences (Figure 4a,b).

### 2.4 Adipose tissue SUV_mean_ is correlated with BMI in breast cancer patients

**FIGURE 5:**
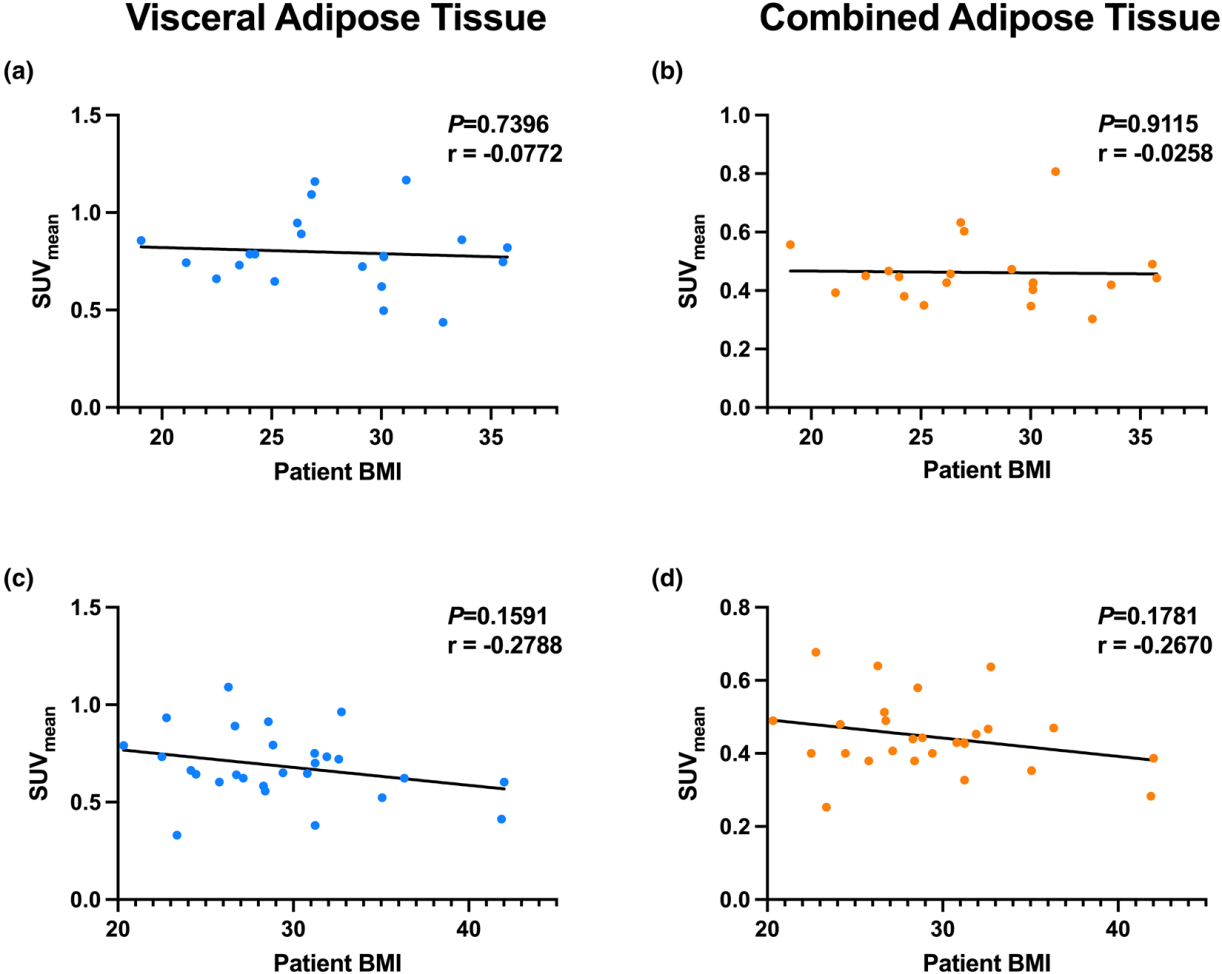
BMI shows stronger correlation with SUV_mean_ in postmenopausal patients. (a, b) Premenopausal patients (*N* = 20) show a weak, non‐statistically significant negative correlation between BMI and Visceral Adipose Tissue SUV_mean_ (*r* = −0.077) and an even weaker relationship between BMI and Combined Adipose Tissue SUV_mean_ (*r* = −0.0258). (c, d) While still not statistically significant, postmenopausal patients (*N* = 28) show a stronger negative correlation between adipose tissue SUV_mean_ and patient BMI for both VAT (*r* = −0.2788) and SAT (*r* = −0.2670).

Across both menopausal subgroups, we evaluated how VAT and SAT tissue metabolic activity, specifically through SUV_mean_, relates to patient BMI. In premenopausal patients, there was no significant correlation between BMI and SUV_mean_ in either visceral adipose tissue VAT or SAT. In VAT, patients displayed no observable trend (*r* = −0.0772), while in SAT, the correlation was even weaker (*r* = −0.0258), suggesting minimal metabolic variation by BMI in younger patients (Figure 5a,b).

**FIGURE 6:**
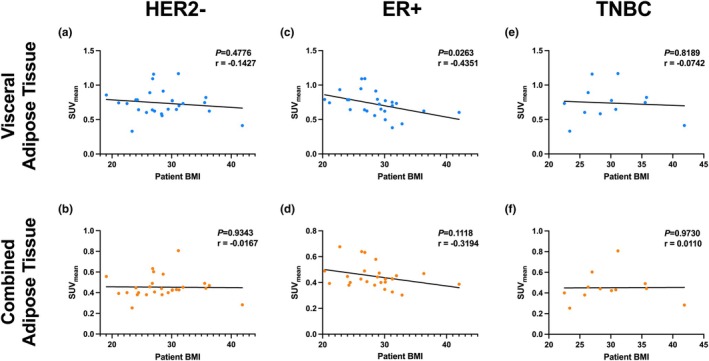
BMI shows a moderate correlation with SUV_mean_ of adipose tissue in ER+ tumors; however, the correlation is insignificant in other breast cancer subtypes. (a, b) Patients with HER2‐negative tumors show no correlations of adipose tissue SUV_mean_ with BMI. (c, d) ER+ tumors show a statistically significant negative relationship between BMI and SUV_mean_, with an *r* = −0.435 in visceral adipose tissue, and also a negative trend in combined adipose tissue–both with minimal outliers. (e, f) TNBC shows no relationship between BMI and SUV_mean_ in adipose tissue types with *r* = 0.0005 and *r* = 0.0001 in visceral adipose and combined adipose tissue, respectively.

In contrast, postmenopausal patients showed a somewhat stronger trend toward an inverse relationship between BMI and SUV_mean_ in both fat compartments. While not statistically significant, both VAT (*r* = −0.2788) and SAT (*r* = −0.2670) trended toward lower metabolic activity in patients with higher BMI (Figure 5c–d). These trends imply that adipose metabolic function may decline with increasing BMI more evidently in older patients. When further stratifying patients by tumor receptor status, we again observe a significant inverse relationship between VAT SUV_mean_ and BMI in patients with ER+ tumors (*r* = −0.4351), suggesting that ER+ tumors may be particularly influenced by adipose metabolic status (Figure 6c). Patients with HER2‐negative tumors also showed a weak negative correlation (*r* = −0.1427), though not significant, while in TNBC patients, there was no relationship observed between BMI and VAT SUV_mean_ (*r* = −0.0742), suggesting the absence of a relationship between visceral metabolic activity and BMI in patients with triple‐negative tumors (Figure 6a,e). For SAT, similar trends were seen but to a lesser degree. ER+ patients showed a modest, non‐significant negative correlation (*r* = −0.3194), while HER2‐negative and TNBC groups showed essentially no correlation (Figure 6b,d,f).

### 2.5 Clinical variables are correlated with adipose tissue SUV and tumor proliferation metrics

**FIGURE 7:**
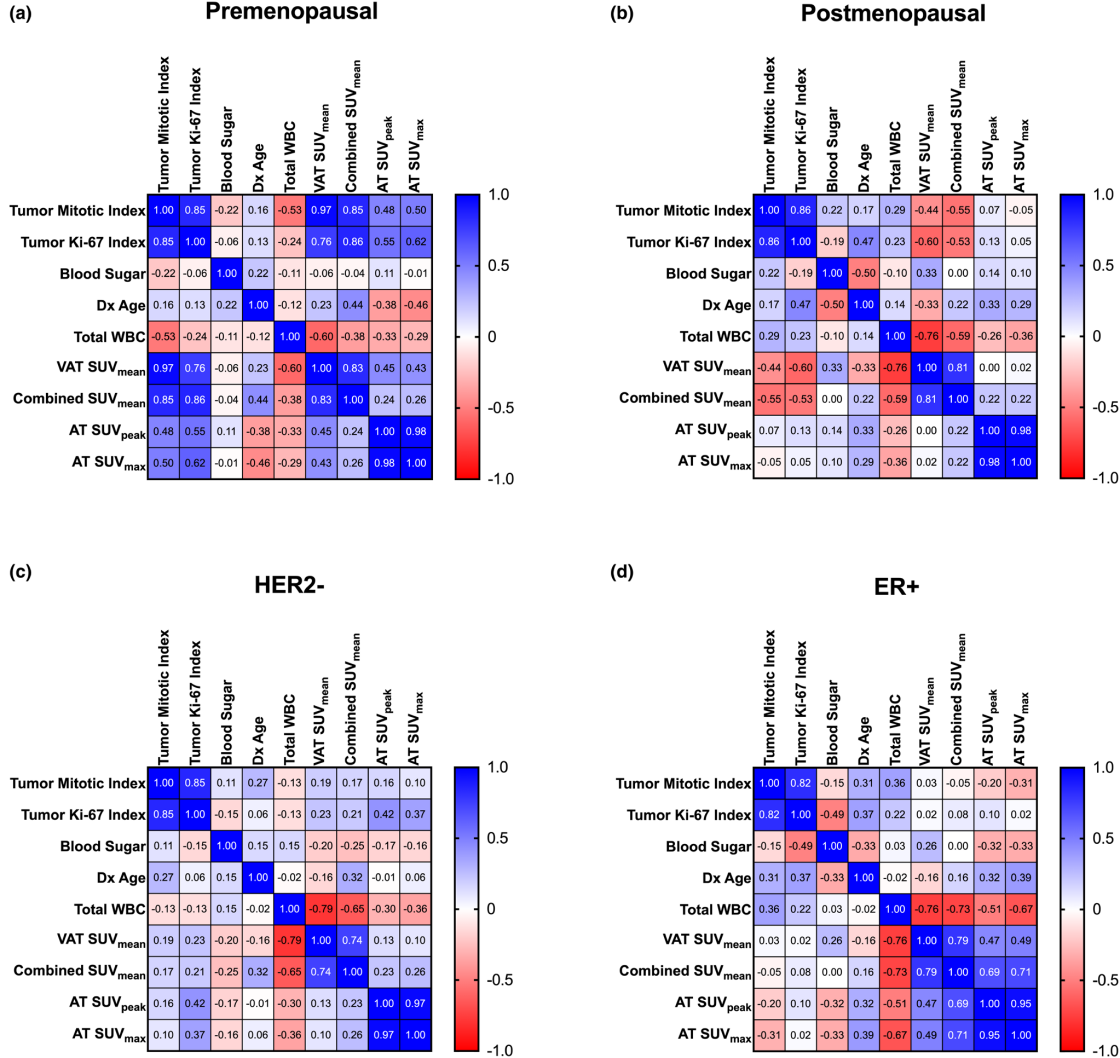
The relationship between SUV_mean_ adipose tissue types and tumor proliferation markers differs between postmenopausal and premenopausal patients, but is similar between patients with HER2‐negative and ER+ tumors. (a) In premenopausal patients (*N* = 8), VAT and SAT SUV_mean_ shows strong positive correlations with tumor mitotic and tumor Ki‐67 index (*r*: 0.76–0.97), AT SUV_peak_ and SUV_max_ also show positive relationships with tumor proliferation metrics (*r*: 0.48–0.62). (b) Postmenopausal patients (*N* = 8) exhibit the inverse relationship, as VAT and SUV_mean_ show negative correlations with tumor mitotic and tumor Ki‐67 index (*r*: −0.60 to −0.44), AT SUV_peak_ and SUV_max_ show no relationship with tumor proliferation metrics. (c) In patients with HER2‐negative tumors, clinical variables and proliferation markers show weak relationships across most variables; however, VAT and SAT SUV_mean_ is strongly negatively associated with total WBC (*r* = −0.79, *r* = −0.65). VAT and SAT SUV_mean_ are weakly positively correlated with AT SUV_peak_ and SUV_max_. (d) Patients with ER+ tumors also have weak correlations between clinical variables and proliferation markers, but show a strong negative correlation of VAT and SUV_mean_ with total WBC as well (*r* = −0.76, *r* = −0.73); however, VAT and SAT SUV_mean_ is more strongly positively correlated with AT SUV_peak_ and SUV_max_ (*r*: 0.49–0.71).

In premenopausal patients, adipose SUV metrics were positively correlated with tumor proliferation markers (mitotic index and Ki‐67 index), showcasing a potential link between increased adipose metabolic activity and more proliferative tumors. Notably, VAT SUV_mean_ showed a particularly strong correlation with mitotic index (*r* = 0.97), and SAT SUV_mean_ also tracked closely with both tumor indices (*r* > 0.85) (Figure 7a). In contrast, in postmenopausal patients, adipose SUV metrics were inversely correlated with tumor proliferation metrics. VAT SUV_mean_ showed moderate negative associations with both mitotic index (*r* = −0.44) and Ki‐67 (*r* = −0.60), and SAT SUV followed a similar trend. Furthermore, WBC count was also strongly negatively correlated with VAT SUV_mean_ (*r* = −0.76), suggesting that higher systemic inflammation may coincide with reduced adipose metabolic activity (Figure 7b). In HER2‐negative patients, adipose SUV metrics were again tightly correlated with each other but showed minimal association with either tumor proliferation indices or WBC count (Figure 7c). However, in ER+ patients, adipose SUV values were quite negatively correlated with both Ki‐67 and WBC count (Figure 7d).

## 3 DISCUSSION

In this study, we examine the impact of PPARγ, a nuclear receptor that plays a central role in adipocyte differentiation, lipid uptake, and anti‐inflammatory signaling, making it a compelling candidate for mediating the intersection between systemic metabolism and breast cancer outcomes (Chi et al., 2021; Hernandez‐Quiles et al., 2021; Li et al., 2024). Given prior epidemiological and mechanistic evidence linking obesity to a worse breast cancer prognosis in postmenopausal women, this study aimed to clarify whether *PPARγ* expression and adipose tissue proliferative activity, as measured by ^18^F‐FLT PET‐CT SUV metrics, could help explain this association across tumor subtypes and menopausal states. Our findings reinforce that high *PPARγ* expression is broadly associated with improved survival, consistent with its previously documented role to promote oxidative metabolism and suppress pro‐inflammatory adipokine signaling in adipose‐rich environments such as the breast (Kim & Ko, 2023; Sun et al., 2021). Interestingly, this relationship was particularly robust in premenopausal patients and in those with hormone receptor‐positive tumors, especially the ER+ subtype (Figure 2). This supports prior literature suggesting that estrogen‐responsive tumors are metabolically entwined with adipose‐derived cues such as leptin, adiponectin, and free fatty acids—all of which are modulated by PPARγ activity (Andrade et al., 2021; Bhardwaj et al., 2019). Conversely, in the TNBC subgroup, high PPARγ expression trended toward poorer long‐term survival (Figure 2g–i). Although not statistically significant and based on limited sample size, this pattern may reflect subtype‐specific metabolic rewiring. Similar mechanisms have been described in hepatocellular carcinoma, where PPARγ activity can be co‐opted by aggressive tumors to support immune evasion in nutrient‐scarce microenvironments (Xiong et al., 2023).

We contextualized these transcriptomic findings with PET‐CT imaging analysis of ^18^F‐FLT uptake in adipose tissue. While average proliferative activity (SUV_mean_) in visceral and combined fat was relatively consistent across tumor subtypes and menopausal status, SUV_peak_ and SUV_max_ were significantly elevated in patients with TNBC or ER‐ tumors as compared to those with hormone receptor‐positive/HER2‐positive or ER+ subtypes, respectively (Figure 4). These higher SUV metrics may reflect enhanced adipose tissue turnover or local proliferative responses, which could possibly be linked to inflammatory or immune cell infiltration–both of which are known to shape the metabolic microenvironment in aggressive breast cancers (Ali et al., 2016; Danforth, 2021; Dieci et al., 2021). The consistently (but modestly) lower adipose SUV values in ER+ tumors could reflect PPARγ‐mediated metabolic inactivity or lipid storage programs that dampen inflammatory signaling and limit local proliferative stimuli.

Interestingly, our data also showed that the inverse relationship between adiposity (measured via BMI) and adipose SUV_mean_ was only evident in postmenopausal patients, not in premenopausal patients (Figure 5). These results align with prior studies, which show that after menopause, declining estrogen levels lead to greater visceral fat accumulation and adipose dysfunction, characterized by reduced mitochondrial activity, increased hypoxia, and elevated secretion of pro‐inflammatory cytokines such as IL‐6 and TNF‐α (Malutan et al., 2014). PPARγ is known to suppress many of these pathways by promoting fatty acid uptake and oxidation. Still, its expression and efficacy may be attenuated by age‐related shifts in the adipose transcriptome (Ulrich‐Lai & Ryan, 2013). These findings suggest that the menopausal state and adipose tissue composition modulate the prognostic relevance of metabolic genes, such as PPARγ, and may inform stratification strategies for patients.

Additionally, when we examined adipose metabolism in relation to tumor proliferation (via Ki‐67 and mitotic index), we found a notable dichotomy. In premenopausal patients, SUV_mean_ in both VAT and SAT positively correlated with tumor proliferation, suggesting metabolic crosstalk or shared growth‐promoting signals between fat and tumor tissue (Figure 7a). In contrast, this relationship reversed in postmenopausal patients, where higher SUV_mean_ was associated with lower proliferation (Figure 7b). This may reflect the altered secretory profile of postmenopausal adipose tissue, where hypertrophy and macrophage infiltration lead to the release of lipotoxic factors rather than growth‐promoting ones (Kawai et al., 2021). Alternatively, it may suggest that tumor aggressiveness in older patients becomes decoupled from adipose metabolism and is driven more by tumor factors or immune suppression.

Moreover, across both HER2‐negative and ER+ subgroups, we found strong negative correlations between WBC count and VAT SUV_mean_ (Figure 7c,d), reinforcing a potential link between systemic inflammation and suppressed adipose metabolic function in breast cancer across these subtypes. These findings are consistent with prior work demonstrating that chronic inflammation impairs adipocyte oxidative capacity and to some capacity also promotes insulin resistance—both of which have been implicated in tumor progression and poorer clinical outcomes (Mili et al., 2021). PPARγ activation has been shown to reduce WBC‐mediated inflammation in metabolic disease and certain cancers, raising the question of whether targeted PPARγ agonism could improve outcomes by restoring adipose function and mitigating paracrine tumor support. Relevant to all comparisons, when data from larger cohorts are available, interaction modeling to understand the prognostic relevance of PPARγ expression as it relates to tumor subtype and clinical characteristics will be of great interest. We acknowledge that not adjusting for multiple comparisons is a limitation, but we believe that it is in the best interest of data transparency, because given the limitations on available samples to analyze, adjusting for multiple comparisons could limit the discovery of biologically relevant trends in smaller subgroups.

Finally, although BMI is widely used as a proxy for adiposity, it is increasingly clear that visceral adiposity and functional fat metabolism are better predictors of metabolic risk and of cancer risk and mortality than BMI alone (De Lorenzo et al., 2013; Khan et al., 2023; Leitner et al., 2022; Mili et al., 2021). This is underscored by our finding that TNBC patients showed no correlation between BMI and adipose SUV (Figure 6), supporting the idea that BMI may mask underlying metabolic dysregulation in aggressive subtypes. It is important to consider the limitations of our study, including reliance on retrospective, heterogeneous datasets, the small TNBC imaging cohort which limits generalizability, the absence of validation in an independent cohort, and restriction of PET‐CT analysis to L3/L4 slices, which may not fully capture whole‐body adipose metabolism. Future studies should incorporate prospective validation in independent cohorts, functional assays to clarify mechanism, multimodal integration with proteomic and lipidomic biomarkers (e.g., visceral fat volume, leptin/adiponectin ratios, and tissue‐level lipidomics) to more comprehensively define these dynamics, and utilize a full multilevel regression framework between clinical variables once there is a higher sample size. Taken together, our study highlights the utility of integrating transcriptomic and metabolic imaging data to better understand the heterogeneity of breast cancer prognosis. *PPARγ* appears to be most prognostically relevant in ER+ tumors, especially in premenopausal patients, where its metabolic effects are most intact. By contrast, its protective role diminishes with age and may even invert in TNBC, where tumors can exploit lipid metabolism for growth, resulting in an opposite trend. Further studies evaluating pharmacologic PPARγ agonists such as rosiglitazone, particularly in metabolically vulnerable breast cancer subtypes, are warranted to test its translational relevance in breast cancer from our findings, as was done in a single, small study which aimed to examine tumor biomarkers rather than outcomes (Yee et al., 2007). Ultimately, this work reinforces the complex but actionable relationship between systemic metabolism, hormonal status, and tumor behavior in breast cancer.

## 4 CONCLUSION

In summary, while limited by data availability, our RNA transcriptomic and imaging analyses reveal that high *PPARγ* expression may be broadly associated with improved breast cancer survival, particularly in ER+ and premenopausal patients, which we hypothesize is facilitated through enhanced lipid oxidative metabolism and anti‐inflammatory regulation (across tumor types). This protective effect diminishes after menopause, where estrogen loss disrupts adipocyte homeostasis and alters PPARγ‐driven pathways, as indicated by the less distinct comparisons and further represented by the adipose SUV metrics. Notably, in TNBC, PPARγ may be co‐opted to fuel tumor progression, underscoring the subtype‐specific metabolic rewiring that is the focus of this study. Together, these data establish *PPARγ* expression as a context‐dependent regulator of breast cancer outcome, linking adipose metabolism, tumor hormonal expression, and tumor behavior—supporting its potential as a biomarker and therapeutic target in metabolically vulnerable subgroups, paving the way for more personalized medicine.

## 5 METHODS

### 5.1 TCGA‐BRCA RNAseq analyses

After confirming strong expression of *PPARγ* in breast tissue using the GTEx bulk tissue expression portal (Figure S1C), as shown in the literature and previous studies, we utilized the publicly available IlluminaHiSeqRNAseq data of patients from the TCGA Breast Cancer (BRCA) data set which we downloaded from the UCSC Xena site; it can be found here: https://xenabrowser.net/datapages/?cohort=TCGA%20Breast%20Cancer%20(BRCA)&removeHub=https%3A%2F%2Fxena.treehouse.gi.ucsc.edu%3A443. The gene expression data were provided as log_2_(RSEM normalized counts + 1), where RSEM normalization adjusts for gene length and sequencing depth to allow for gene expression comparisons across samples; all cutoffs are reported in these units. We then processed this data using a custom in‐house R script to generate Kaplan–Meier survival plots and associated statistics.

The dataset initially contained RNA sequencing data from 1218 patients; however, only primary tumor samples were retained, and duplicate aliquots were collapsed into a single sample per patient, leaving 1101 unique patients. Clinical metadata were joined to the expression matrix, and overall survival (OS) information was curated from TCGA (retaining patients with available OS time and event data). After matching to patients with valid PPARγ expression values, 1094 patients remained for analysis. Expression of PPARγ was rounded to three decimal places and for each analysis, patients were stratified into two groups using a fixed cutoff at  the cohort's median. For the entire cohort the patients were stratified: a low‐expression group (<6.903, *n* = 548) and a high‐expression group (≥6.903, *n* = 546). Kaplan–Meier survival analysis was then performed for overall survival up to 8605 days (~23.5 years) of follow‐up, with log‐rank tests and Cox proportional hazards regression used to estimate significance and hazard ratios.

After the initial analyses, we incorporated menopausal status as a phenotypic variable from the clinical matrix in the R pipeline for the same patient set. Menopausal status was categorized as premenopausal, postmenopausal, or perimenopausal. These patients were then further stratified for two analyses; patients with high *PPARγ* expression as described above were selected for one cohort and the other cohort contained data from patients with low *PPARγ* expression For both of these analyses any patients with an indeterminate menopausal status were removed, which resulted in 492 patients with a high expression that had an available menopausal status, and 479 with low expression with an available menopausal status. For those with high *PPARγ* expression, 125 were premenopausal, 345 were postmenopausal, and 22 were perimenopausal; for those with low *PPARγ* expression 103 were premenopausal, 359 were postmenopausal, and 17 were perimenopausal. These samples were again visualized with cutoffs set to 6000+ days for both groups after initial diagnosis, to visualize long‐term survivability relationships.

After performing the analyses non‐specifically on all patients with primary tumors, the data were divided by tumor type: ER+ or HER2‐negative tumors were then studied, which were chosen as a phenotypic parameter from the clinical matrix. After filtering, there were 598 patients with ER+ tumors and 649 patients with HER2‐negative tumors. It is important to note that patients could have both tumor types and that they are not independent of one another. For these initial tumor type‐specific analyses, in the ER+ cohort, there were 300 patients with low *PPARγ* expression (<6.866 ) and there were 298 with high expression (≥6.866), and in the HER2‐negative cohort, there were 325 patients with low expression (<6.803) and 324 with high expression (≥6.803). The cutoffs for *PPARγ* expression dividing patients differ across conditions, as it was determined to be the median of the specific cohort. Patients were then divided by their menopausal status among their breast cancer cohort, and it was visualized if *PPARγ* gene expression showed differences between groups of different menopausal statuses. For the ER+ cohort, there were 135 total premenopausal patients, and 68 had low *PPARγ* gene expression (<7.228) and 67 had high expression (≥7.228); there were 390 postmenopausal patients with ER+ tumors, and 195 had low *PPARγ* expression (<6.630) and 195 had high *PPARγ* expression (≥6.630). For the HER2‐negative cohort, there were 152 premenopausal patients, and 76 had low *PPARγ* expression (<7.140) and the 76 others had high expression (≥7.140) and there were 418 postmenopausal patients, with 209 of low expression (<6.791) and 209 with high expression (≥6.791). For the TNBC cohort, there were 133 patients overall with available *PPARγ* gene expression data; of these, 62 had low *PPARγ* gene expression (<6.700) and 61 had high expression (≥6.700). In the premenopausal subgroup, there were 34 patients: 17 had low expression (<6.741), and 17 had high expression (≥6.741). Among the 69 postmenopausal patients, 35 had low *PPARγ* expression (<6.720), and 34 had high expression (≥6.720). Since these stratified tumor‐subtype specific analyses had a much lower sample size, it is important to note that these analyses were intended to be exploratory rather than confirmatory.

### 5.2 Human patient image analyses

In this study, we perform image analysis on PET/CT scans from 69 of the 90 patients from an open‐source data repository from The Cancer Imaging Archive (TCIA)–all patients with menopausal status and scans at the L3/L4 level were included in this study. The dataset can be found here: https://wiki.cancerimagingarchive.net/pages/viewpage.action?pageId=30671268. These patients were part of a clinical trial (ACRIN‐6888), all data was de‐identified and written consent was provided from the patients during the time of the study. This data was originally approved for public release by the University of Arkansas for Medical Sciences (UAMS) Institutional Review Board (IRB #205568)–all participants provided informed consent for data sharing through TCIA; however, we did not have access to the specific procedures or documentation of the consent process. As the dataset was fully de‐identified and its use pre‐approved under the original IRB protocol, no additional IRB review was required for our secondary analysis. Furthermore, 48 out of 69 of the patients studied had their height and weight available, allowing for their BMI to be calculated, and 68 of the patients had their tumor receptor status (i.e., HER2 and ER) available, allowing for stratification during the analyses. Each of the patients had scans ranging from their first visit until post‐treatment–in order to minimize the effect of the clinical trial itself, and to focus the study on breast cancer cohorts only the first available scans were used for the patient, as treatment for breast cancer can skew overall patient metabolism and tumor proliferation (Peck et al., 2015). Overall from the 69 patients selected, 32 were premenopausal and 37 were postmenopausal. Furthermore, there were 38 patients with ER+ tumors and 41 patients with HER2‐negative tumors–it is important to note that a patient could have both an ER+ and HER2‐negative tumor, as they are not mutually exclusive since the receptors are independent of each other. Patients that were negative for ER, HER2, and PR receptors were selected as part of the triple‐negative cohort, which included 17 patients.

Images from each of the patients were uploaded to Fiji ImageJ and using the Brown Fat ROI tool within the PET‐CT viewer, we were able to analyze the scans. Since adipose tissue (visceral and subcutaneous) was being studied, we assessed all scans at the L3 or L4 vertebral level, where the tumor is located, and which is the standard landmark for assessing these scans. In particular, to only quantify the standard uptake value of ^18^F‐FLT, we set the CT criteria to −190 to −30 Hounsfield Units (HU) to selectively segment adipose tissue, and not other tissue or bone in the surrounding regions. We then drew fixed‐volume spheres around the area of interest and were able to calculate the lean body mass‐corrected SUV values (including SUV_mean_, SUV_peak_, and SUV_max_), and for each patient, we performed analysis on 3 separate continuous slices in the region. SUV_mean_ represents the average uptake across the entire region of interest. SUV_peak_ corresponds to the average uptake within the most metabolically active small sub‐volume and SUV_max_ reflects the single highest voxel value, which together show measures of areas of highly concentrated tracer distribution.

### 5.3 Statistical analysis

In this study, we rely on previously available data for both the TCGA‐BRCA RNA‐seq data of 1094 patients and the PET‐CT image analyses from 69 breast cancer patients. Survival outcomes were first evaluated by generating Kaplan–Meier plots to visualize the cumulative probability of overall survival across time by gene expression. The plots also included 95% confidence intervals around the survival curves as shaded bands, to provide an estimate of uncertainty at each timepoint and to show overlap with the other cohort/group with which it was being compared. A “number at risk” table was placed below each Kaplan–Meier plot to indicate how many patients remained under observation at successive intervals. To formally test whether survival distributions differed between groups, we performed log‐rank tests. This non‐parametric test compares observed versus expected survival events across strata and produces a *p*‐value for differences in survival. The test statistic and corresponding significance levels were calculated in R using the survival package, and have been annotated directly onto the figure. It is important to note that due to the reliance on previously collected data, some cohorts, including the TNBC cohort which included only 133 total patients, were underpowered. This analysis serves to be more of an exploratory analysis rather than confirmatory. Since we decided to use OS as our metric of an event, to have death as a ground‐truth label of an event for stronger clinical significance, there was a slight decrease in power due to that: resulting in 71% overall power of the entire cohort, which was even lower in the stratified analyses.

Hazard ratios (HRs) were calculated using Cox proportional hazards regression, which estimates the relative hazard of death between groups while making no assumptions about the baseline hazard function. For each comparison, the model generated a hazard ratio alongside its 95% confidence interval. A HR <1 indicated a protective association with longer survival, whereas an HR greater than 1 suggested increased risk. Finally, Cox models were utilized to study clinical covariates, including age, menopausal status, and receptor status (ER, PR, HER2). This allowed adjustment of potential confounding variables, and testing whether gene expression remained independently associated with survival outcomes (Table S1).

From the 69 patients included for image analysis, 32 were premenopausal and 37 were postmenopausal. Among the 48 patients with complete height and weight data, body mass index (BMI) ranged from 19.04 to 42.02 kg/m^2^, with 10 patients classified as healthy weight (18–24.9 kg/m^2^), 18 as overweight (24.9–29.9 kg/m^2^), and 20 as obese (≥30 kg/m^2^). The mean BMI was 28.64 kg/m^2^ with a standard deviation of 5.03 kg/m^2^. Standardized uptake values (SUV) were then calculated for adipose tissue, and patients were stratified into cohorts based on menopausal status and tumor type. Comparisons were performed in GraphPad Prism version 10.5.0, and to assess differences in SUV distributions (e.g., between premenopausal vs. postmenopausal patients or by tumor subtype), we employed the Mann–Whitney U test, a non‐parametric alternative to the independent *t*‐test that does not assume normality. In addition to reporting *p*‐values, we calculated rank‐biserial correlation coefficients as an effect size measure, which quantifies the magnitude and direction of the difference between two independent groups (Table S2). This allowed us to interpret by both statistical significance  and practical effect size.

We also performed Spearman's rank correlations to examine relationships between SUV values and available laboratory or clinical parameters, including blood glucose, Ki‐67 proliferation index, and receptor status. Only patients with complete lab values were included in these analyses, yielding 16 patients stratified by menopausal status (8 premenopausal, 8 postmenopausal), 13 HER2‐negative patients, and 10 ER+ patients. Due to limited sample size, the TNBC subgroup (*n* = 4) was excluded. Within these correlation matrices, both correlation coefficients (Figure 7) and their associated *p*‐values were reported (Table S3), allowing us to assess both the strength and the statistical significance of associations across clinical and imaging variables.

For this study, statistical significance was indicated by *p*‐values <0.05, and marginally significant results have *p*‐values >0.05 but <0.10.

## AUTHOR CONTRIBUTIONS

The study was conceptualized by Aditya Shah and Rachel J. Perry. Data collection and analysis were performed by Aditya Shah, Katie Liu, Ryan Liu, Gautham Ramshankar, and Curtis J. Perry. The figures and the initial draft of the manuscript were generated by Aditya Shah, and all authors reviewed, edited, and approved the manuscript. Rachel J. Perry supervised and obtained funding for the study.

## FUNDING INFORMATION

Support was provided by the National Institutes of Health: R37 CA258261‐01A1 (to Rachel J. Perry).

## CONFLICT OF INTEREST STATEMENT

None of the authors have any conflicts of interest relevant to this study.

## Supporting information


**Figure S1.** (A) Bioinformatics workflow for TCGA‐RNA seq analyses. (B) Example PET‐CT scan at L3‐L4 level with annotated regions. (C) PPARγ is highly expressed in breast tissue and adipose tissue.
**Table S1.**Multivariable Cox proportional hazards regression analysis of overall survival by PParγ expression and clinical covariates.
**Table S2.** Rank‐biserial effect sizes (*r*) for adipose uptake metrics across clinical subgroups (Menopause, HER2, ER, TNBC).
**Table S3.** *p*‐values from Spearman correlation analyses of adipose uptake metrics across clinical subgroups.

## Data Availability

All data analyzed in this manuscript is publicly available at the links in the corresponding parts of the Methods section; they are also listed in this section. The TCGA‐BRCA RNA‐seq dataset (gene expression) utilized for analysis can be found at the following Xenabrowser link: https://xenabrowser.net/datapages/?cohort=TCGA%20Breast%20Cancer%20(BRCA)&removeHub=https%3A%2F%2Fxena.treehouse.gi.ucsc.edu%3A443. The PET‐CT scan images can be found in The Cancer Imaging Archive at the following link: https://www.cancerimagingarchive.net/collection/acrin‐flt‐breast/. Any code utilized for the analysis is available from the corresponding author upon request.
