# Supplementary material for: Interactions among tumor subtype, PPARγ expression, and adipose proliferation shape outcomes in breast cancer
Source: Physiol Rep. 2025 Nov 14;13(21):e70649. doi: 10.14814/phy2.70649 (PMC12617248; doi:10.14814/phy2.70649)
Supplement: Supplementary file 1 — Figure S1. (A) Bioinformatics workflow for TCGA‐RNA seq analyses. (B) Example PET‐CT scan at L3‐L4 level with annotated regions. (C) PPARγ is highly expressed in breast tissue and adipose tissue. Table S1.Multivariable Cox proportional hazards regression analysis of overall survival by PParγ expression and clinical covariates. Table S2. Rank‐biserial effect sizes (r) for adipose uptake metrics across clinical subgroups (Menopause, HER2, ER, TNBC). Table S3. p‐values from Spearman correlation analyses of adipose uptake metrics across clinical subgroups. [file PHY2-13-e70649-s001.docx]

**Supplemental Data**


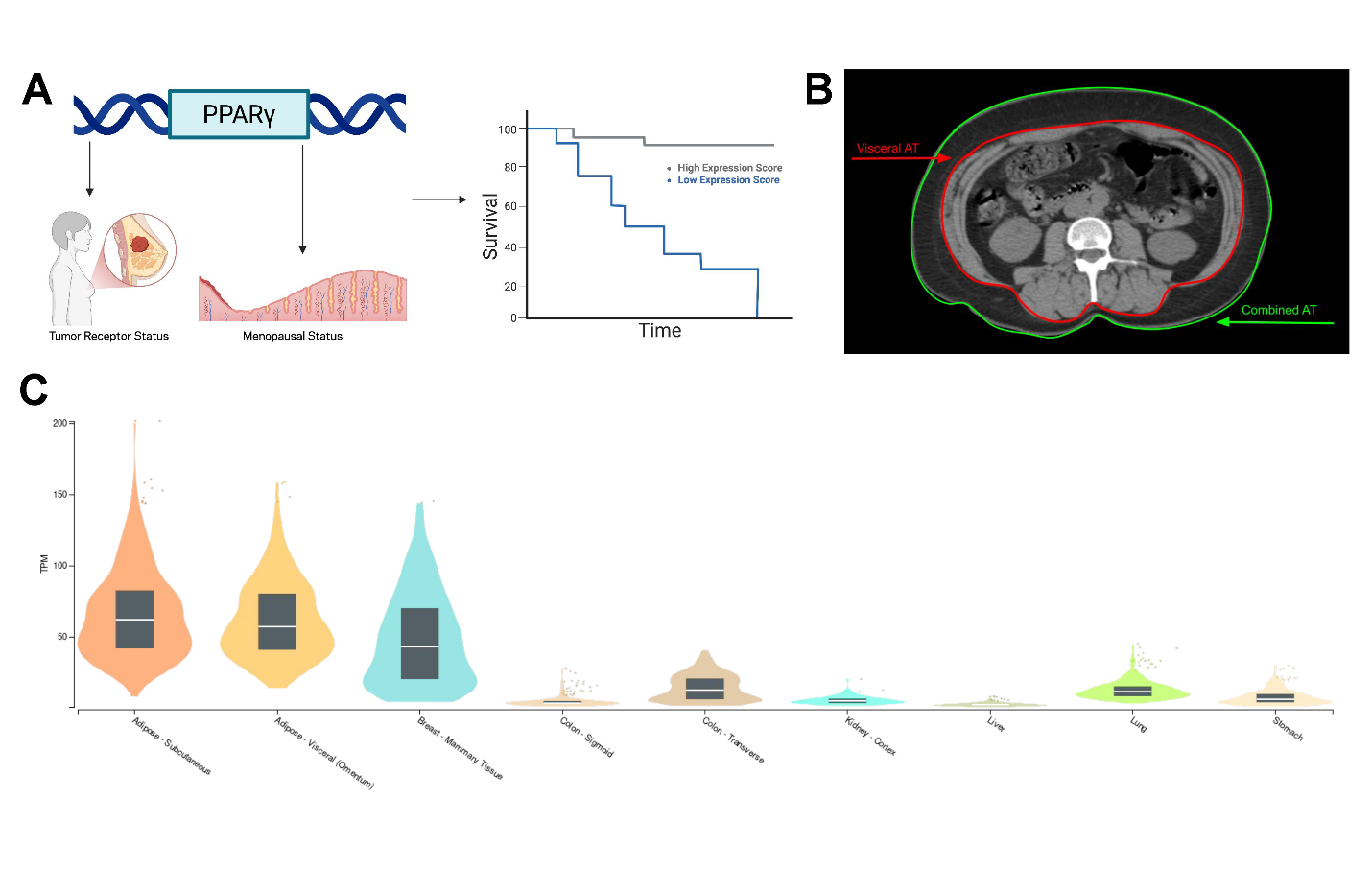


**Figure S1**: **(A)** Bioinformatics workflow for TCGA-RNA seq analyses. **(B)** Example PET-CT scan at L3-L4 level with annotated regions. **(C)** PPARγ is highly expressed in breast tissue and adipose tissue.

| **Variable** | **HR** | **CI_low** | **CI_high** | **P-Value** |
| --- | --- | --- | --- | --- |
| **PPARG >= 6.903 (vs <)** | 0.7299325551 | 0.4440779562 | 1.199792801 | 0.2143936952 |
| **Age (per year)** | 1.047226795 | 1.020743398 | 1.07439731 | 0.0004140327093 |
| **Postmenopausal (vs pre)** | 0.7424577423 | 0.3188663972 | 1.728760083 | 0.489842579 |
| **ER positive (vs negative)** | 1.322019379 | 0.6079426563 | 2.874835678 | 0.4812304517 |
| **PR positive (vs negative)** | 0.6255197352 | 0.320373391 | 1.221309104 | 0.1693380114 |
| **HER2 positive (vs negative)** | 0.8638056822 | 0.4050987301 | 1.841921984 | 0.7047191887 |

**Table S1.** Multivariable Cox proportional hazards regression analysis of overall survival by PPARG expression and clinical covariates.

| **Variable** | **Visceral r** | **Combined r** | **Peak r** | **Max r** |
| --- | --- | --- | --- | --- |
| **Menopause** | 0.22 | 0.024 | 0.088 | 0.111 |
| **HER2** | 0.098 | 0.023 | 0.034 | 0.07 |
| **ER** | 0.113 | 0.134 | 0.428 | 0.407 |
| **TNBC** | 0.015 | 0.021 | 0.329 | 0.368 |

**Table S2.** Rank-biserial effect sizes (r) for adipose uptake metrics across clinical subgroups (Menopause, HER2, ER, TNBC).

| **Premenopausal Patients** | | | | | | | | | |
| --- | --- | --- | --- | --- | --- | --- | --- | --- | --- |
|  | **Tumor mitotic index** | **Tumor Ki-67 index** | **Blood Sugar** | **Age at Diagnosis** | **Total WBC** | **VAT SUV_mean_** | **Combined SUV_mean_** | **AT SUV_peak_** | **AT SUV_max_** |
| Tumor Mitotic Index | 1 | 0.01151 | 0.58958 | 0.70838 | 0.18462 | 0.00060 | 0.011508 | 0.231151 | 0.20893 |
| Tumor Ki-67 Index | 0.01151 | 1 | 0.89792 | 0.76052 | 0.58214 | 0.03676 | 0.01071 | 0.17098 | 0.114980 |
| Blood Sugar | 0.58958 | 0.89791 | 1 | 0.59166 | 0.80813 | 0.89792 | 0.94355 | 0.80813 | 0.98978 |
| Age at Diagnosis | 0.70838 | 0.76052 | 0.59167 | 1 | 0.77966 | 0.58810 | 0.27341 | 0.34583 | 0.25804 |
| Total WBC | 0.18462 | 0.58214 | 0.80813 | 0.77966 | 1 | 0.13229 | 0.35987 | 0.42788 | 0.50079 |
| VAT SUV_mean_ | 0.00060 | 0.03676 | 0.89792 | 0.58810 | 0.13229 | 1 | 0.01538 | 0.26746 | 0.29921 |
| Combined SUV_mean_ | 0.01151 | 0.01071 | 0.94355 | 0.27341 | 0.35987 | 0.01538 | 1 | 0.58214 | 0.53641 |
| AT SUV_peak_ | 0.23115 | 0.17098 | 0.80813 | 0.34583 | 0.42788 | 0.26746 | 0.58214 | 1 | 3.97E-04 |
| AT SUV_max_ | 0.20893 | 0.11498 | 0.98978 | 0.25804 | 0.50079 | 0.29921 | 0.53641 | 3.97E-04 | 1 |
| **Postmenopausal Patients** | | | | | | | | | |
|  | **Tumor mitotic index** | **Tumor Ki-67 index** | **Blood Sugar** | **Age at Diagnosis** | **Total WBC** | **VAT SUV_mean_** | **Combined SUV_mean_** | **AT SUV_peak_** | **AT SUV_max_** |
| Tumor Mitotic Index | 1 | 0.01131 | 0.61667 | 0.69970 | 0.48274 | 0.27917 | 0.17396 | 0.88720 | 0.92768 |
| Tumor Ki-67 Index | 0.01131 | 1 | 0.64663 | 0.24643 | 0.58591 | 0.12480 | 0.18884 | 0.75833 | 0.91587 |
| Blood Sugar | 0.61667 | 0.64663 | 1 | 0.21617 | 0.84013 | 0.42788 | 1 | 0.75203 | 0.84013 |
| Age at Diagnosis | 0.69970 | 0.24643 | 0.21617 | 1 | 0.75203 | 0.42788 | 0.61726 | 0.42788 | 0.50079 |
| Total WBC | 0.48274 | 0.58591 | 0.84013 | 0.75203 | 1 | 0.03676 | 0.14107 | 0.53641 | 0.38938 |
| VAT SUV_mean_ | 0.27917 | 0.12480 | 0.42788 | 0.42788 | 0.03676 | 1 | 0.02173 | 1 | 0.97679 |
| Combined SUV_mean_ | 0.17396 | 0.18884 | 1 | 0.61726 | 0.14107 | 0.02173 | 1 | 0.61726 | 0.61726 |
| AT SUV_peak_ | 0.88720 | 0.75833 | 0.75203 | 0.42788 | 0.53641 | 1 | 0.61726 | 1 | 3.97E-04 |
| AT SUV_max_ | 0.92768 | 0.91587 | 0.84013 | 0.50079 | 0.38938 | 0.97679 | 0.61726 | 3.97E-04 | 1 |
| **Patients with HER2- Tumors** | | | | | | | | | |
|  | **Tumor mitotic index** | **Tumor Ki-67 index** | **Blood Sugar** | **Age at Diagnosis** | **Total WBC** | **VAT SUV_mean_** | **Combined SUV_mean_** | **AT SUV_peak_** | **AT SUV_max_** |
| Tumor Mitotic Index | 1 | 0.00054 | 0.71756 | 0.36520 | 0.66190 | 0.52841 | 0.57975 | 0.60554 | 0.75710 |
| Tumor Ki-67 Index | 0.00054 | 1 | 0.61134 | 0.85814 | 0.66608 | 0.44997 | 0.48602 | 0.15259 | 0.21793 |
| Blood Sugar | 0.71756 | 0.61140 | 1 | 0.62452 | 0.61358 | 0.51332 | 0.40242 | 0.56875 | 0.59401 |
| Age at Diagnosis | 0.36520 | 0.85814 | 0.62452 | 1 | 0.94392 | 0.59996 | 0.28614 | 0.97446 | 0.83833 |
| Total WBC | 0.66190 | 0.66608 | 0.61358 | 0.94392 | 1 | 0.00183 | 0.01835 | 0.31636 | 0.22456 |
| VAT SUV_mean_ | 0.52841 | 0.44997 | 0.51332 | 0.59996 | 0.00183 | 1 | 0.00491 | 0.66918 | 0.75064 |
| Combined SUV_mean_ | 0.57975 | 0.48602 | 0.40242 | 0.28614 | 0.01835 | 0.00491 | 1 | 0.45301 | 0.39354 |
| AT SUV_peak_ | 0.60554 | 0.15259 | 0.56875 | 0.97446 | 0.31636 | 0.66918 | 0.45301 | 1 | 3.84E-07 |
| AT SUV_max_ | 0.75710 | 0.21793 | 0.59401 | 0.83833 | 0.22456 | 0.75064 | 0.39354 | 3.84E-07 | 1 |
| **Patients with ER+ Tumors** | | | | | | | | | |
|  | **Tumor mitotic index** | **Tumor Ki-67 index** | **Blood Sugar** | **Age at Diagnosis** | **Total WBC** | **VAT SUV_mean_** | **Combined SUV_mean_** | **AT SUV_peak_** | **AT SUV_max_** |
| Tumor Mitotic Index |  | 0.82164 | -0.15053 | 0.30640 | 0.36490 | 0.02501 | -0.04704 | -0.20010 | -0.30640 |
| Tumor Ki-67 Index | 0.82164 | 1 | -0.49390 | 0.37082 | 0.21713 | 0.01824 | 0.07622 | 0.10334 | 0.01824 |
| Blood Sugar | -0.15053 | -0.49390 | 1 | -0.33435 | 0.03364 | 0.26140 | 0 | -0.31611 | -0.32827 |
| Age at Diagnosis | 0.30640 | 0.37082 | -0.33435 | 1 | -0.01829 | -0.16364 | 0.16413 | 0.32121 | 0.39394 |
| Total WBC | 0.36490 | 0.21713 | 0.03364 | -0.01829 | 1 | -0.75611 | -0.73089 | -0.50611 | -0.67074 |
| VAT SUV_mean_ | 0.02501 | 0.01824 | 0.26140 | -0.16364 | -0.75611 | 1 | 0.79028 | 0.46667 | 0.49091 |
| Combined SUV_mean_ | -0.04704 | 0.07622 | 0 | 0.16413 | -0.73089 | 0.79028 | 1 | 0.69301 | 0.71125 |
| AT SUV_peak_ | -0.20010 | 0.10334 | -0.31611 | 0.32121 | -0.50611 | 0.46667 | 0.69301 | 1 | 0.95152 |
| AT SUV_max_ | -0.30640 | 0.01824 | -0.32827 | 0.39394 | -0.67074 | 0.49091 | 0.71125 | 0.95152 | 1 |

**Table S3**. P-values from Spearman correlation analyses of adipose uptake metrics across clinical subgroups.
